# A modulated fingerprint assisted machine learning method for retrieving elastic moduli from resonant ultrasound spectroscopy

**DOI:** 10.1038/s41598-023-33046-w

**Published:** 2023-04-11

**Authors:** Juejing Liu, Xiaodong Zhao, Ke Zhao, Vitaliy G. Goncharov, Jerome Delhommelle, Jian Lin, Xiaofeng Guo

**Affiliations:** 1 Department of Chemistry, Washington State University, Pullman, WA 99164 USA; 2 Alexandra Navrotsky Institute for Experimental Thermodynamics, Washington State University, Pullman, WA 99164 USA; 3 School of Mechanical and Materials Engineering, Washington State University, Pullman, WA 99164 USA; 4 Department of Chemistry, University of Massachusetts, Lowell, MA 01854 USA; 5 School of Nuclear Science and Technology, Xi’an Jiaotong University, Xi’an, 710049 Shaanxi China

**Keywords:** Materials science, Techniques and instrumentation

## Abstract

We used deep-learning-based models to automatically obtain elastic moduli from resonant ultrasound spectroscopy (RUS) spectra, which conventionally require user intervention of published analysis codes. By strategically converting theoretical RUS spectra into their modulated fingerprints and using them as a dataset to train neural network models, we obtained models that successfully predicted both elastic moduli from theoretical test spectra of an isotropic material and from a measured steel RUS spectrum with up to 9.6% missing resonances. We further trained modulated fingerprint-based models to resolve RUS spectra from yttrium–aluminum-garnet (YAG) ceramic samples with three elastic moduli. The resulting models were capable of retrieving all three elastic moduli from spectra with a maximum of 26% missing frequencies. In summary, our modulated fingerprint method is an efficient tool to transform raw spectroscopy data and train neural network models with high accuracy and resistance to spectra distortion.

## Introduction

Elastic moduli are important thermodynamic parameters of extended solid-state phases, which are also closely correlated with structural features, such as lattice symmetry, grain boundary, and material defects^1–7^. Such elastic information provides the foundation for studying the functionality of materials, including superconductors, bionic materials, and semiconductors^7–9^. Conventionally, elastic moduli are measured by static and dynamic methods. Measuring static moduli requires stretching bulk samples by a universal testing machine (UTM, widely used in materials industry) or a dynamic mechanical analysis (DMA, specialized for analyzing polymer)^10–12^. In the dynamic method, samples are vibrated by waves, *e.g.*, ultrasonic wave. One can then calculate the dynamic elastic moduli by using the velocity of wave, density, and Poisson’s ratio of materials^13,14^.

Resonant ultrasound spectroscopy (RUS) obtains the elastic moduli from natural resonance frequencies, yielding all elastic moduli from a single measurement^15,16^. By knowing all elastic moduli, one can calculate different thermodynamic parameters, *e.g.*, heat capacity, Debye temperature^17,18^. RUS is an affordable and accurate method to obtain such thermodynamic properties compared with thermochemical methods (*e.g.*, calorimetry) or spectroscopy methods (*e.g.*, X-ray absorption spectroscopy, XAS). RUS has been commonly used to characterize new materials, including high entropy alloys and ceramic-based superconductors^7,9,15,16^. However, there are still two major drawbacks for implementing RUS. First, to derive moduli from a RUS spectrum, one needs initial guesses of elastic moduli, which ideally are close to their true values. Nevertheless, it is sometime challenging to make the guessed moduli closed to the actual ones, especially when the investigated material is novel without previous knowledge. Bad values of initial guesses may lead fitting to diverge or converge on an incorrect local minimum^15^. Second, the experimental RUS spectra may contain missing resonance frequencies caused by vibrational nodes at the transducer contact point. This also makes the determination of elastic moduli prone to subjective guessing. In short, using RUS not only requires knowledge of the materials of interest but also some practical experience. Thus, it is necessary to develop new methods to analyze RUS spectra without user intervention.

Herein, we introduced neural network-based deep learning to analyze RUS spectrum and automatically retrieve elastic moduli. As a supervised machine learning method, deep learning has advantages for modeling complicated non-linear relationships due to its distinctive “layers of nodes” structure. A deep learning algorithm can unveil the relationship between features and labels if sufficient data is fed for the training of neural network model^19–21^. After proper training, the resulting models are capable of independently predicting a label (*e.g*., elastic modulus) from a not-yet-seen feature (e.g., experimental spectrum). The intent is to use this algorithm to minimize human intervention that is conventionally required for deriving elastic moduli from RUS. Previously, similar deep learning methods were successfully applied to analyze spectra from various characterization techniques, including XAS, infrared (IR) and UV–Vis spectroscopy, X-ray diffraction (XRD), and fluorescence^22–28^. For instance, different models were developed to identify the structure of nanoparticles from the X-ray absorption fine structure (XAFS) spectra^26,29,30^. Because of the characteristics of these spectroscopy methods, it is possible to use the raw or slightly preprocessed (*e.g*. subtracting nano particle spectra from bulk material spectrum)^26^, computed or experimental spectra to train the models.

For applying deep learning methods to the analysis of RUS spectra, we identified several challenges. In previous deep learning studies of XRD and XAS, the training datasets consisted of hundreds of thousands measured spectra (XRD, obtained from open-source databases) or computed spectra (XAS, generated by *Feff code*)^26,28,29^. These spectra are essentially two-dimensional data (e.g., with useful information from both diffraction angle/energy and intensity). Therefore, only minimal preprocessing is needed. In contrast, the computed RUS spectra are lists of resonances frequencies corresponding to a specified set of elastic tensor dimension, and density of samples—amplitude is not possible to compute because of the extreme variability of transducer contact and displacements^15,16,31^. Therefore, it is unfeasible to efficiently train models without strategically preprocessing such one-dimensional data. This issue can even become more complicated, considering experimental errors in sample dimensions, shape, and morphology. For instance, shifting and missing modes are common^15^. Missing modes are less predictable during experiments, in terms of whether missing modes occur, and if so, which modes are missing^15,32^. In contrast, the shifting of modes can be attributed to an asymmetrical distortion of crystallographic structure and thus, to changes in elastic moduli^33,34^. In total, a strategically designed data preprocessing method is important to convert raw data (both computed and experimental) into an easy-to-train format. To our knowledge, there has only been so far a single deep learning work for the analysis of RUS spectra that revealed the hidden order transition temperature (T_HO_) of URu_2_Si_2_^35^, where the model successfully classified the hidden order of T_HO_ (one-component or two-component).

Previous successful applications of deep learning to spectroscopy, and particularly RUS, have motivated us to pursue models that can directly obtain elastic moduli from RUS spectra with tolerance in imperfect experimental RUS spectra containing shifting or missing modes. Inspired by image classification and recognition studies, we modulated the raw computed RUS spectra into an image-like format as input for training a neural network (NN). We first trained a regression model to recognize two elastic moduli (C11 and C12) for a steel cylinder. After proper training process, the resulting NN model automatically recognized the two elastic moduli from a test dataset containing computed RUS spectra and an experimentally measured spectrum, both containing randomly missing resonant frequencies. Lastly, we successfully applied NN models on retrieving the elastic moduli from both isotropic and anisotropic cerium-doped yttrium aluminum garnet (Ce:YAG) ceramic samples, demonstrating the potential of NN models for broader inorganic material systems.

## Method

### Software libraires

All software libraries we used in this study were listed below: TensorFlow2, NumPy, Pandas^36–40^. We developed software to compute RUS spectra based on Lagrange minimization^41^. The python packages NumPy and Pandas were used to preprocess the raw theoretical spectra (described below). TensorFlow2 was used to construct the NN and produce the models. The TensorFlow2 code was written using Python 3.8.

### Theoretical RUS dataset

The theoretical resonant modes were obtained by locating the stationary points of the Lagrangian, which is given by Eq. (1)^42^:

1 $$L = \mathop \smallint \limits_{V}^{{}} \left( {KE - PE} \right)dV$$

where V is the sample’s volume, KE and PE are respectively the kinetic and potential energy density as shown in Eq. (2) and (3):

2 $$KE = \frac{1}{2}\mathop \sum \limits_{i} \rho \omega^{2} u_{i}^{2}$$

3 $$PE = \frac{1}{2}\mathop \sum \limits_{i.j.k.l} c_{ijkl} \frac{{\partial u_{i} }}{{\partial x_{j} }}\frac{{\partial u_{k} }}{{\partial x_{l} }}$$

where ρ is the sample density, ω is the angular frequency, u_i_ is the i_th_ component of displacement vector, and C_ijkl_ is the stiffness tensor. The displacement vector is expanded by the Eq. (4):

4 $$u_{i} = \mathop \sum \limits_{\alpha } a_{i\alpha } \varphi_{\alpha } \left( {x,y,z} \right)e^{\omega t}$$

where a_iα_ is the expansion coefficients and ${\varphi }_{\alpha }\left(x,y,z\right)$ is the basis function, which is represented by Legendre polynomials in this work. By substituting the displacement vector into the Lagrangian, the stationary points of the first derivative of the Lagrangian are determined by the eigenvalues as shown in Eq. (5):

5 $$\omega^{2} Ea = \Gamma a$$

where a is the motion assembled from the basis set, E is the kinetic energy term and Γ is the elastic energy term.

Theoretical resonance modes were obtained by varying the elastic constants in the energetic term. Generated datasets are used to train and validate the NN model (Table S1). For steel, the C11 and C12 range were 170–370 GPa and 30–130 GPa in 1.0 GPa increments. For the three Ce:YAG datasets with 0.0025%, 0.01%, and 1% Ce in terms of weight, three elastic moduli ranges, C11, C12, and C44, were 270 to 370 GPa, 60 to 160 GPa, and 60 to 160 GPa in 5 GPa increments, respectively. Sample diameter and mass were fixed during the computed spectra generation. Robustness of the inverse method was also tested against experimental uncertainties. To simulate possible imperfect experimental data, resonance shifting of modes were intentionally introduced into the dataset by adding random frequency shifting of − 1.5% to + 1.5% and. By doing so, we obtained 7 spectra from every single computed spectrum generated by Eq. (5). The randomly missing modes were simulated by a dropout layer, which will be discussed in NN model architecture section. Every dataset of steel cylinder and Ce:YAG samples contained totally about 140000 computed spectra. The data distributed eventually in these datasets. There is no specified range inside the dataset showing either a higher or a lower data density than other ranges. We divided this dataset into three subsets, namely train, validation, and test datasets with a ratio of 5:1:1.

### Measured spectra

All measured RUS spectra used in this study were taken with an ACE RUS-008 model instrument^31^. The steel spectrum was obtained from a cylinder standard sample made in accordance with ASME B 18.8.2, hardened ground machined with a core hardness of RC 47–58 and a minimum case hardness of RC 60. Surface finish is about 0.2 microns maximum and dimensional accuracy of about 25 microns for length and 2 microns for diameter. The three Ce:YAG experimental spectra were obtained from the ceramic samples prepared in our previous study^7^. Detailed sample parameters and experimental configurations were listed in Table S2. Specifically, the frequency range for collecting RUS spectrum from steel cylinder standard sample was described by the operation manual of the ACE RUS-008 instrument in the calibration section. While the frequency range of collecting spectra from the three Ce:YAG samples was determined based on our pervious study^7^.

### Data preprocessing and input format

Three data preprocessing methods were proposed and studied, direct input, derivative input, and modulated fingerprint. In general, a computed RUS spectrum consisted of a series of resonant frequencies, *f*_*n*_, corresponding to specified elastic moduli, mass, and diameter of sample. We defined the computed spectra as the features and the corresponding elastic moduli as the labels. The sample diameter and mass were fixed and they are parameters easy to measure. In direct input method, we did not heavily preprocess the computed RUS spectra. We obtained a sequence of the first 64 resonant frequencies from every computed spectrum, *f*_*1*_ to *f*_*64*_, with a single increase order. These sequences and corresponding elastic moduli were utilized to train the NN models. Empirically, in RUS data processing, the first 25 peaks were sufficient to derive elastic moduli^7^.

In the derivative method, the frequency differences between two nearby modes, *Δf*_*n*_, were used as the features. The *Δf*_*n*_ was obtained by Eq. (6).

6 $$\Delta f_{n} = \, f_{n + 1} {-} \, f_{n}$$

where *f*_*n*+*1*_ and *f*_*n*_ were two nearby resonant frequencies. To obtain the *Δf*_*n*_ sequence with *n* between 1 to 64, the first 65 resonant frequencies, *f*_*1*_ to *f*_*65*_, from a computed spectrum were used. This process is equivalent of calculating the first derivative between resonant frequencies values versus order of frequencies.

The *f*_*n*_ and *Δf*_*n*_ in every sequence of features of direct input and derivative-input-based datasets were rescaled between 0 and 1 by considering the maximum and minimum frequencies or different of frequencies values in each of the datasets (Eq. 7 and 8).

7 $$f_{n, \, rescaled} = \, \left( {f_{n} - \, f_{min} } \right)/\left( {f_{max} - \, f_{min} } \right)$$

8 $$\Delta f_{n, \, rescaled} = \, \left( {\Delta f_{n,} - \, \Delta f_{min} } \right)/\left( {\Delta f_{max} - \, \Delta f_{min} } \right)$$

where *f*_*min*_ and *f*_*max*_ were the minimum and maximum frequencies, while *Δf*_*min*_ and *Δf*_*max*_ were the minimum and maximum difference of frequencies in the whole dataset.

Modulated fingerprint method has a different approach for preprocessing computed RUS spectra (see Fig. S1). Firstly, the frequency range of interest (*F*) in a computed RUS spectrum is evenly divided into specified number of bins (*B*), from 64 (8 × 8) to 1024 (32 × 32). Every bin shares the same length (*l*) in terms of range of frequency (see Eq. 9).

9 $$l \, = \, F/B$$

We then count the number of modes (*N*_*f*_) inside each bin with assigning its number as *b*_*n*_. The range of *n* is from 1 to *B* (see Eq. 10).

10 $$b_{n} = \, N_{{f, \, [l \, \times \, \left( {n - 1} \right), \, l \, \times \, n]}}$$

The sequence of *b*_*n*_ from 1 to *B* corresponds to a square image with *B*^*1/2*^ × *B*^*1/2*^ resolution, where *b*_*n*_ represents the grayscale of every pixel inside the image.

The elastic moduli, C11, C12, and C44, in direct input, derivative input, and modulated fingerprint input were also rescaled between 0 and 1by Eq. (11).

11 $$C_{i,j, \, rescaled} = \, \left( {C_{i,j} - \, C_{i,j, \, min} } \right)/\left( {C_{i,j, \, max} - \, C_{i,j, \, min} } \right)$$

### NN model architecture

The NN used in this study consisted of four to five layers: an input layer, a dropout layer, one or two hidden dense layers, and an output layer. The features, e.g., sequences of rescaled *f*_*n*_, *Δf*_*n*_, or *b*_*n*_, were first introduced into the NN by the input layer. For the direct input and derivative input, the number of nodes at the input layer was 64 corresponding to 64 *f*_*n*_ or *Δf*_*n*_ in the sequences. For the modulated fingerprint, seven different configurations were used, 64, 144, 256, 400, 576, 784, and 1024 corresponding to *B*, the number of bins. A dropout layer was assigned after the input layer. This layer randomly sets exact to 10% of input nodes as 0 at every training step. Therefore, this dropout layer was useful to enhance the resistance of models against missing frequencies. We then built models either with one hidden dense layer (with 48, 64, 80, 96 and 112 nodes inside, respectively) or two hidden dense layers (64 nodes for the first hidden layer and 16 nodes for the second hidden layer). TensorFlow optimized the weight and bias parameters in every node in the dense layer(s) during training process. The goal of such optimization was to automatically build a function to calculate elastic moduli from input spectra. Lastly, we assigned two nodes for the steel dataset or three nodes for the Ce:YAG dataset at the output layer. The nodes in the output layer were to show the rescaled elastic moduli, *e.g.*, C11, C12, and C44, for the input spectra. This is because steel is an isotropic material containing only two elastic moduli (C11, and C12). For the YAG material, it may exhibit an anisotropic behavior when doping with Ce. Therefore, it has maximum three elastic moduli (C11, C12, and C44). After reversing the rescaling process, the human readable elastic moduli were obtained.

### Training notes

A conventional desktop computer (processer: Intel I5-9400F, graphical processing unit: Nvidia RTX 3070, memory: 16 GB) was used to train all models. We used Adam optimizer to train the NN models and mean square error (MSE) as the loss function. Adam optimizer is an efficient gradient descent algorithm for training NN models^43^. During training, the NN determines the relation between features and labels (in this case, computed RUS spectra and elastic moduli) through an iterative process by optimizing the parameters of every node in the dense layers. The MSE loss function shows the mean square error between the true elastic moduli and calculated elastic moduli. Therefore, the MSE is the metric for measuring the accuracy of the fitting (see Eq. 12).

12 $$MSE = \left( \frac{1}{n} \right) \times \mathop \sum \limits_{i = 1}^{n} \left( {C_{i, real,rescaled} - C_{i, retrived,rescaled } } \right)^{2}$$

Adam optimizer guides the iterative process towards convergence by minimizing MSE. The function for calculating the elastic moduli from computed RUS spectra was obtained from the deep learning model. An example equation for obtaining C11 from model with one hidden layer is shown below.

13 $$C11_{norm. } = \left[ {b_{2} + \mathop \sum \limits_{j = 1}^{{n_{2} }} W\left( {2, 3,j,1} \right)f_{2} \left[ {\mathop \sum \limits_{i = 1}^{{n_{1} }} W\left( {1, 2, i,j} \right)G_{i} )} \right]} \right]$$

where *f*_*2*_ is the activation function (ReLU in this case), *b*_*2*_ is the bias, *W(k-1, k, i, j)* is the weight matrix between two layers of nodes. *G*_*i*_ is the input nodes^44^. Bias was not applied to the input layer. The number of nodes in the first layer (input layer) and second layer (hidden layer) is *n*_*1*_ and *n*_*2*_. We set the initial learning rate for the Adam optimizer as 1 × 10^–3^. During the iterative process, the learning rate was then divided by 5 until 1 × 10^–6^ if MSE did not decrease after 10 epochs. The batch size of the training dataset was set to 60. Therefore, the NN adjusted all the internal parameters one time after seeing every 60 spectra during each training epoch. The training dataset was shuffled, and it was repeated 4 times during every epoch. Although the model learned every piece of training data 4 times, but the data was slightly different due to the dropout of 10% of nodes.

About 100,000 and 20,000 computed RUS spectra were used in training and validation of the model. As the sample parameters, *e.g.*, density and diameter, were fixed during dataset generation, the resulting model was corresponded with one specified sample. With this configuration, the training time was relatively short even using a conventional computer (about 6 h). In contrast, although training a universal model to predict elastic moduli from all different samples is theoretically plausible, the training dataset of such a model would consist of millions, if not billions, of computed RUS spectra. It is hard to train such a model without using a significant amount of computational power. Therefore, we believe that training a model for a specific sample is more feasible than training a universal model.

### Training and evaluation of different NN models

To study the effect of preprocessing methods on the accuracy of models, we trained the NN models with the computed steel cylinder dataset with 2048 epochs. To evaluate the accuracy of models, we randomly chose 128 spectra from the test dataset, which we did not use during training and validation of NN models. Then we utilized models to obtain elastic moduli from the 128 spectra. We fed the models with both intact spectra and spectra with 6% of missing modes. Mean absolute percentage error (MAPE) was adopted to compare the true and retrieved elastic moduli from the models (see Eq. 14). C11, C12, were compared individually.

14 $$MAPE = 100\% \times \frac{1}{n}\mathop \sum \limits_{i = 1}^{n} \left| {\frac{{C_{i,real} - C_{i,retrieved} }}{{C_{i,real} }}} \right|$$

We prefer using MAPE to evaluate accuracy of models because it directly shows the difference between ground truth and prediction of elastic moduli.

While studying the effect of fingerprint resolution on the accuracy of models, we trained all three models with 512 epochs with the computed steel cylinder dataset. Then we utilized the resulting models to retrieve elastic moduli from the test dataset. To evaluate the stability of the model against missing modes, we observed the MAPEs of C11 and C12 from spectra with up to 9.6% of missing frequencies. We further input the experimental steel cylinder spectrum into the best model to evaluate the accuracy of the model when solving experimental spectrum. Like the computed spectra in the test dataset, we randomly removed up to 12.5% of frequencies to simulate missing modes.

We also trained three models to retrieve elastic moduli from RUS experimental spectra of three Ce:YAG ceramic samples with 2048 epochs. Since the three samples had different diameters and mass, we generated different computed RUS spectra datasets corresponding to the samples. During the human resolving, we noticed that these experimental spectra intrinsically contain missing and shifting of modes^7^. We then directly compared the difference between the human resolved elastic moduli to the retrieved elastic moduli from NN models.

## Results and discussion

**Figure 1:**
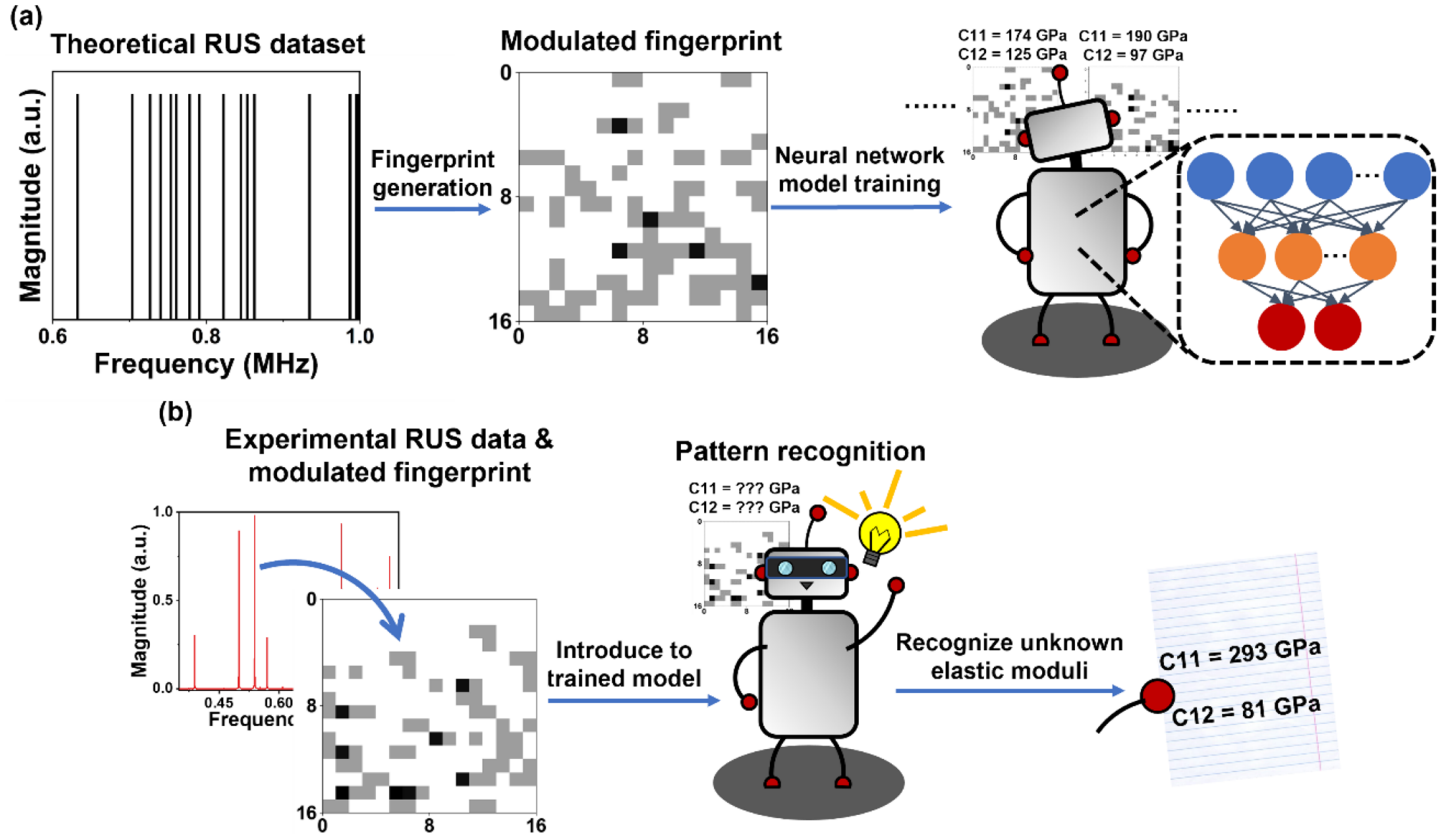
Illustration of training neural network (NN) to recognize elastic moduli by feeding modulated fingerprints corresponding to theoretical RUS spectra (**a**). Using trained model to obtain elastic moduli from an unseen experimental RUS spectrum (**b**).

Data preprocessing is crucial for combining deep learning and materials science. Proper data preprocessing methods optimize the training dataset and therefore improve the accuracy of the resulting NN models. Here, we reported three different data preprocessing methods (direct input, derivative input, and modulated fingerprint) to optimize theoretical RUS spectra for training of NN models to obtain elastic moduli. The modulated fingerprint is the only method in this study to promote the NN models recognizing patterns between computed steel RUS spectra and two elastic moduli (C11 and C12, see Fig. 1a). The resulting model correctly recognized C11 and C12 from both synthetic RUS data that were not used in the training, and experimental RUS data of a steel cylinder, which are also robust in dealing with randomly removed modes (up to 12.5% compared to total modes in data, see Fig. 1b). We further trained NN models based on modulated fingerprint to analyze experimental RUS spectra from Ce-doped yttrium aluminum garnet (Ce:YAG) samples to retrieve three elastic moduli (C11, C12, C44), which also turned to be successfully despite mode missing.

### Comparison of three data preprocessing methods for accuracy and robustness

**Figure 2:**
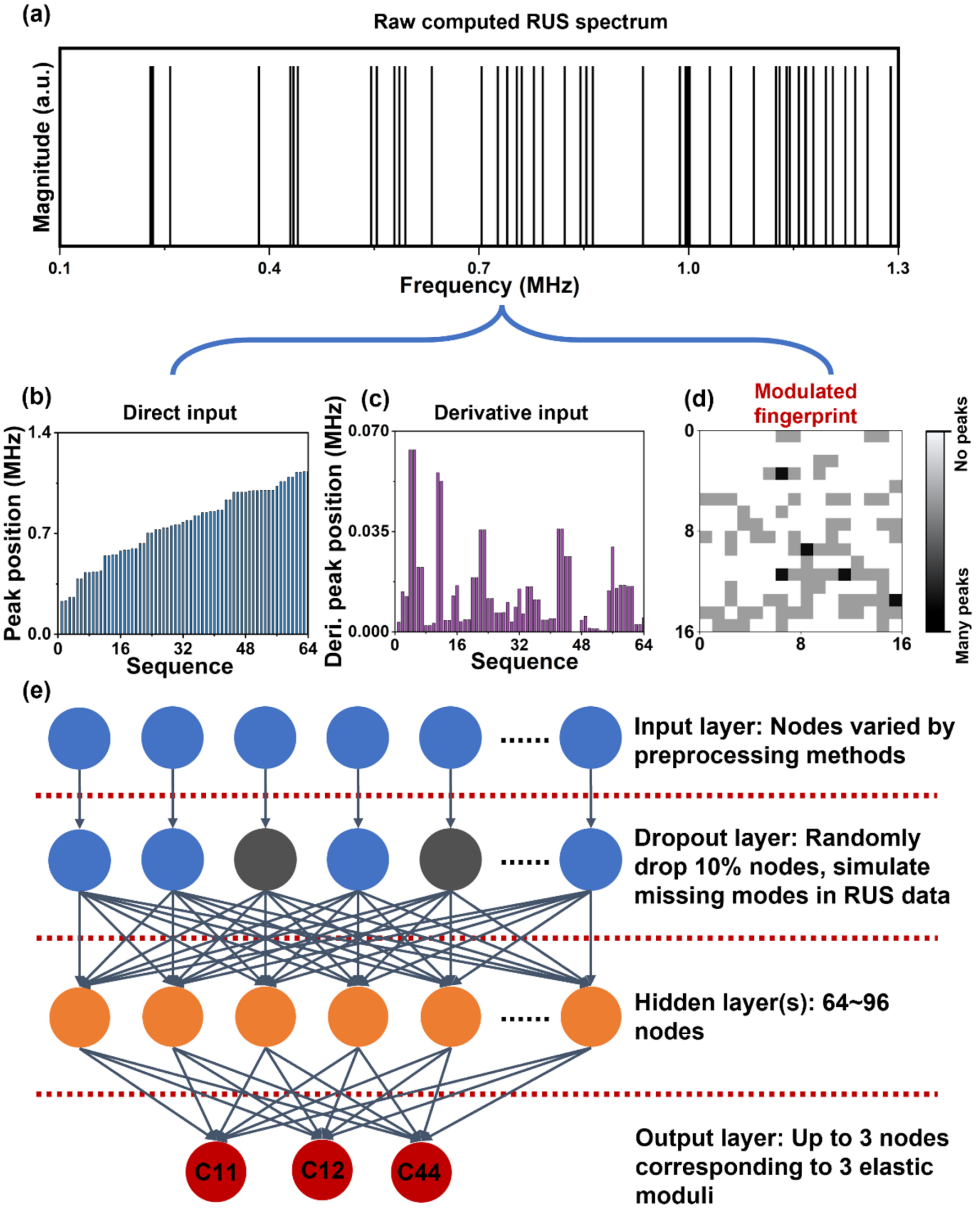
Influence of data preprocessing methods on accuracy of NN models. (**a**) A typical raw computed steel cylinder RUS spectrum (C11: 174.0 GPa, C12: 125.0 GPa). (**b**–**d**) Three methods preprocessing theoretical RUS spectra dataset for neural network training: direct input (**b**), derivative input (**c**), and modulated fingerprint (**d**). (**e**) Structure of neural network utilized in this study.

The training, validation, and test steel RUS spectra dataset with specified C11 and C12 ranges were computed by Lagrange equation (see Table S1 for all parameters we used to compute the dataset). The C11 and C12 range were determined from a combination of past studies on different steel samples and our empirical experience on RUS data analysis^45–47^. In this work, we generated about 20 thousands synthetic RUS spectra in the dataset for training of NN-based models. As one needs to pick the correct combination of C11 and C12 from such a large range of data to solve the experimental steel cylinder data, manual fitting of RUS data is generally slow or impossible. As shown in Fig. 2a, each computed RUS spectrum is a list of potential resonant frequencies corresponding to specified parameters of samples, such as elastic moduli set, spatial parameters, and density^15^. To increase the model accuracy and decrease training time, it is necessary to preprocess this dataset before training NN^26,30,48,49^. When designing the preprocessing methods (direct input, derivative input, and modulated fingerprint), addressing two goals were needed. First, the method should amplify the distinction of features from computed RUS spectra with different elastic moduli. Second, missing frequencies should not significantly change the features from the same computed RUS spectra.

As shown in Fig. 2b, Figs. S2a and b, the direct input method did not distinguish computed spectra with different elastic moduli. This lack may potentially impact the generalization of NN between features (spectra) and labels (elastic moduli). To overcome this issue, it is necessary to enhance the difference among all computed spectra^26^. Compared with direct input, the derivative input distinguishes spectra corresponding to different elastic moduli sets, such as position and amplitude of peaks (see Fig. 2c, Figs. S2c and d). The concept of the derivative of a spectrum is widely adopted for enhancing the signal strength in signal processing^50,51^. However, neither direct input nor derivative input is robust against missing resonant frequencies, which is a common issue in the experimental RUS data^35,52^. This is because the missing resonant frequencies change the sequence of frequencies, and that potentially impacts the accuracy of NN models when retrieving elastic moduli. Inspired by the exceptional image classification ability of NN and relating studies^53,54^, we designed a modulated fingerprint method to preprocess and optimize the computed RUS spectra dataset. This method reduces the RUS data from a list of frequencies into an image-like format and breaks the relationship between the sequence of modes and the mode frequencies. As shown in Fig. 2d, Figs. S2e and f, the modulated fingerprints from spectra corresponding to different elastic moduli are easily distinguishable. Such distinguishable features were beneficial for the training of NN-models. We then generated a modulated fingerprint from a compromised spectrum with 6% missing modes and compared it to the fingerprint image from the original intact spectrum. The two fingerprint images were similar (see Fig. S3). Most of the grey pixels (px) did not change their locations. As a result, most of the features of the spectrum were maintained even after missing several modes.

**Figure 3:**
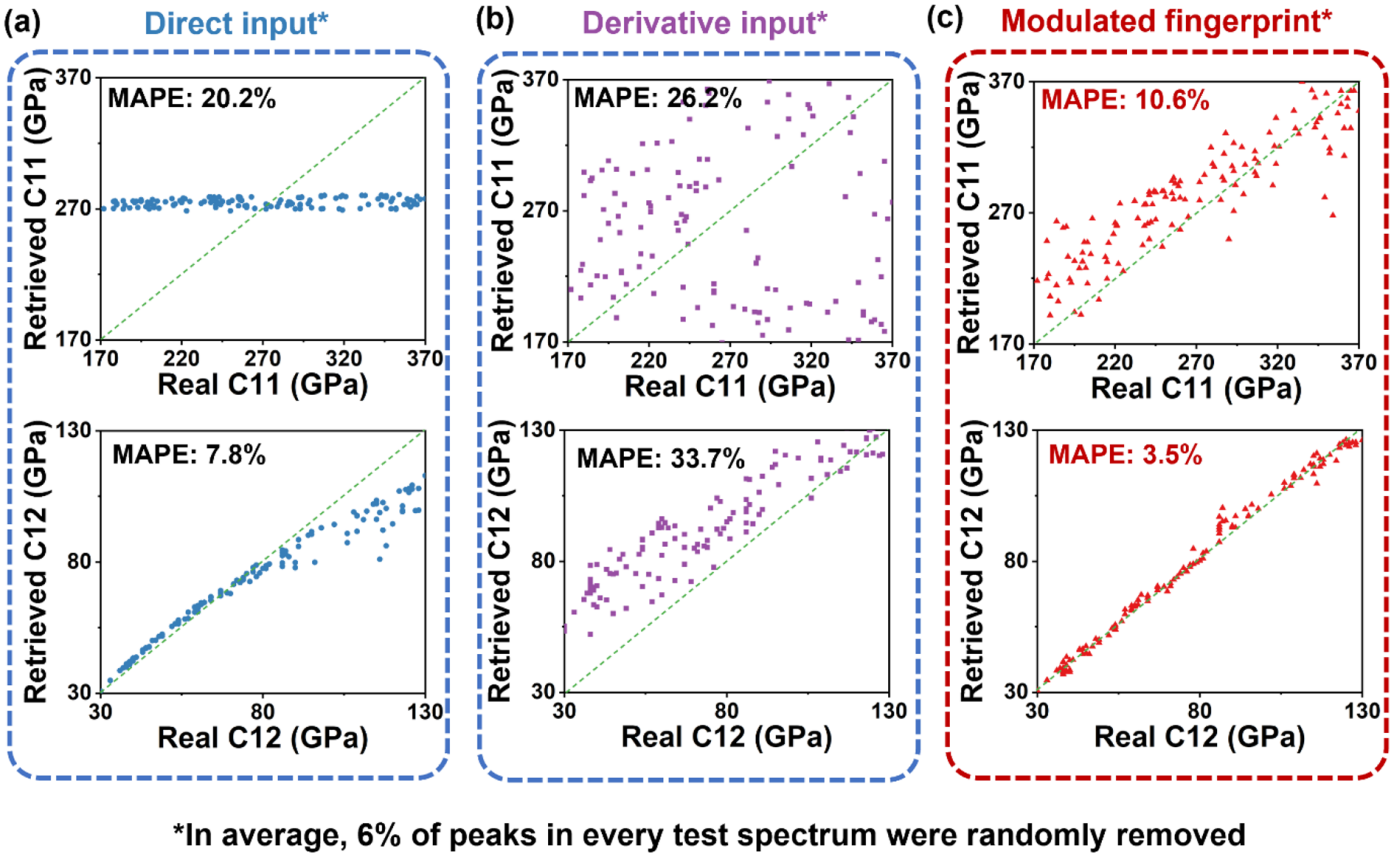
Comparison between real & predict C11 and C12 values from neural network models using direct input (**a**), derivative input (**b**), and modulated fingerprint (**c**) preprocessing methods. We randomly dropped 6% of peaks from every theoretical RUS spectrum in test dataset to simulate missing peaks in RUS experimental spectra.

All three datasets from direct input, derivative input, and modulated fingerprint were used train NN models. This NN was consisted of an input layer, a dropout layer, a dense layer with 64 nodes, and an output layer with 2 nodes corresponding to the 2 elastic moduli (Fig. 2e, also see method section for detailed information). The dropout layer randomly set 10% of the input nodes to 0 during every training epoch to simulate the unintended missing of modes in RUS data. The percentage of dropping out modes was determined by our preliminary attempts after trying many different percentages of dropout rate from 2.5% to 20%. Four datasets were used train models, including the dataset of the steel cylinder and three datasets for three Ce:YAG ceramic samples, which will discuss later. Both the intact spectra (Fig. S4) and defected spectra (Fig. 3) with missing frequencies were used to evaluate the performance of resulting models. The accuracy of these models was evaluated by comparing the true elastic moduli to the predicted elastic moduli with mean absolute percentage error (MAPE). As shown in Fig. S4a and Fig. 3a, direct input-based model did not resolve C11 regardless of feeding intact or 6% mode removed test spectra. The NN model believed the C11 values from all test spectra were about 270.0 GPa, the middle of the C11 range used for training. The direct input-based model resolved the C12 from some of the test spectra with specified C12 range, from 30.0 GPa to 80.0 GPa. Overall, the MAPEs of retrieved C11 and C12 from intact data, 20.1% and 8.4%, and defected data, 20.2% and 7.8%, did not exhibit significant difference. As the direct input does not strategically amplify the difference among spectra, the NN model did not generalize the relation between spectra and elastic moduli. When resolving C11 from intact spectra, derivative input-based model exhibited higher accuracy, 9.7% of MAPE (Fig. S4b). The MAPE of C12, 14.2%, was higher than that of direct input method, but there was a clear trend between true and retrieved value. The retrieved C12 values were always about 7.0 GPa lower than the true C12. However, when feeding the model with spectra containing missing modes, the model failed to recognize any C11 values resulting in a large MAPE, 26.2% (Fig. 3b). The accuracy of C12 resolving was also decreased resulting in a large MAPE at 33.7%. Overall, both models were not capable of handling missing resonant frequencies in RUS spectra.

The modulated fingerprint-based model retrieved the C11 and C12 values from pristine spectra (Fig. S4c) with the highest accuracy among the three methods. The MAPEs were 8.6% and 2.3% when retrieving C11 and C12 from intact spectra. More importantly, the modulated fingerprint-based model maintained similar accuracy when feeding test spectra with missing modes. The resulting MAPEs of C11 and C12 retrieving were 10.6% and 3.5%, respectively (Fig. 3c), demonstrating the success and robustness of the modulated fingerprint method in retrieving elastic moduli from spectra.

### Examining modulated fingerprint based NN structures for accuracy in predicting elastic moduli

To understand the reason why the modulated fingerprint-based model tolerates missing frequencies, we further investigated how the NN models retrieve elastic moduli from spectra. In a nutshell, NN models automatically build functions to calculate elastic moduli from the computed RUS spectra during training process. The different preprocessing methods determine the appearance of the input data to the NN and therefore affect the function building of the NN. The models then use the built functions to retrieve elastic moduli from RUS spectra in the test dataset, which is unseen in the training process. The derivative preprocessing method fed sequences of *Δf*_*n*_ versus elastic moduli to train the NN. The resulted model relies on value and order of *Δf*_*n*_ to calculate the corresponding elastic moduli. Therefore, the integrity of the computed spectra is crucial to generate the sequences of *Δf*_*n*_ correctly represent elastic moduli. Missing even one mode can change all subsequent *Δf*_*n*_ resulting in inaccurate calculation of elastic moduli. In contrast, in the modulated fingerprint method, missing *f*_*n*_ does not significantly change the resulting image-like data (Figs. S3a and b), in which most features, such as location of the grey and dark px, were not changed after randomly removing 6% of modes from the spectrum. Hence, the modulated fingerprint based model exhibited similar accuracy while feeding intact and compromised test spectra.

We then investigated the influence of neural network structures on accuracy. We firstly decreased the number of nodes inside the hidden dense layer from 64 to 48. As shown in Fig. S5a, the resulted model predicted all C11 value as 370 GPa from the spectra in the test dataset, the maximum value inside the steel cylinder dataset, independent of the input spectra. In contrast, the model correctly recognized the values of C12. This result suggests that the hidden layer with only 48 nodes is not capable to recognize relationship between modulated fingerprint and elastic moduli. A hidden layer containing 64 nodes is the minimal requirement to solve RUS spectra. (Fig. S5b). Further increasing the number of nodes (80, 96, and 112 nodes, see Figs. S5c, d, and e) and adding one more hidden layer (see Fig. S5f.), did not result in significantly improved accuracy in terms lowering MAPE, especially for predicting C11 values. Another common practice for training different neural networks with the same dataset is to catch some unseen phenomena by observing the predicting results from all different neural network models and pinpointing the ensemble deviation in the common poor-performance regions^55,56^. However, most neural network models, except the one with a hidden dense layer of 48 nodes, exhibited similar performance among the whole region of interest for both elastic moduli. Thus, we adopted the model with one hidden dense layer with 64 nodes inside because it was the simplest model determining both elastic moduli correctly.

**Figure 4:**
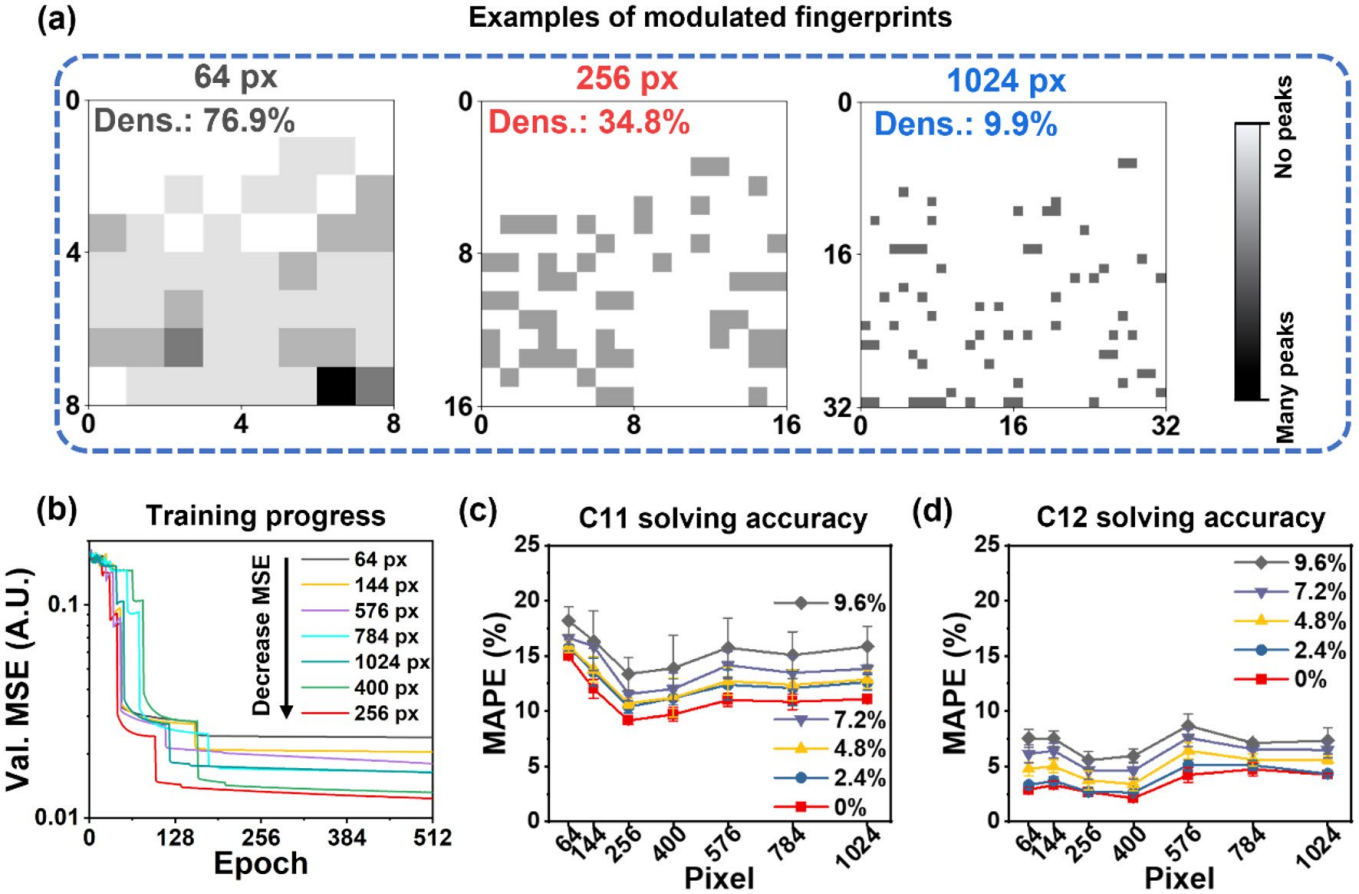
Effect of resolution of modulated fingerprint on the image-like data and accuracy of neural network models. (**a**) Example fingerprints (steel cylinder, C11: 270.0 GPa, C12: 100.0 GPa) with 64 pixels, 256 pixels and 1024 pixels and averaged data density in modulated fingerprints. (**b**) Effect of resolution on the neural network training process. (**c**) and (**d**) Accuracy of models trained by fingerprints with different resolutions while retrieving C11 (**c**) and C12 (**d**) from 128 randomly chosen computed steel cylinder RUS spectra in test dataset. Specified percentage of modes were removed to observe model stability.

In NN model-based image recognition, resolution significantly affects the accuracy of the models^57–59^. We studied seven different resolutions (64, 144, 256, 400, 576, 784, and 1024 px). To analyze the difference among all the resolutions, we defined information density (dens.) as the percentage of pixels containing modes versus all pixels in the fingerprint images. We then averaged this ratio from every spectrum in the training dataset (See Table S3). The information density is gradually decreased with the increasing of resolution of the image-like data. Figure 4a showed three examples with different resolutions (64, 256, and 1024 px) from a computed RUS spectrum of a steel cylinder (C11: 270.0 GPa, C12: 100.0 GPa, diameter: 4.76 mm, height: 3.45 mm, mass: 0.48 g) to highlight the influence of resolution on the input data. In 64 px, the gray and dark blocks occupy most of the image resulting in high information density (76.9%) in the training dataset. As we split the frequency range of interest (0.2 MHz to 1.3 MHz) into only 64 bins, every bin represents a large frequency range (17.2 kHz per bin). Most bins contain one or more peaks. In 256 px, the gray and dark px contain lower information density (34.8%), with each bin occupying 4.3 kHz. In 1024 px, the mode containing px are sparse (information density: 9.9%) because of the increased number of bins and small bin extent (only 1.1 kHz).

We fed all seven datasets to train the NN models to examine the impact of the resolution of the image-like data on accuracy of the resulted models, monitored and validated by their validation mean square error (MSE). As shown in Fig. 4b, 64 px-based NN model struggled to generalize the relationship between features (fingerprints) and labels (elastic moduli), with a highest validation MSE among all models. Increasing the px of the image-like data from 64 px to 144 and 256 px decreases the validation MSE during training. The model trained by 256 px dataset exhibits the lowest MSE in this study. Increasing the resolution to 400 px results in a slightly lower accuracy (mild increase of MSE). All image-like datasets with higher resolutions, including 576, 784 and 1024 px, cause significant increase of validation MSE, which indicates low accuracy in the validation step while training. In total, based on the validation MSE data, the models trained by 256 or 400 px dataset demonstrated better performance than others during training and validation.

The accuracies of the seven models were further examined by solving randomly selected synthetic RUS data in the test dataset. Similar to the validation MSE during training, models trained by dataset with 256 and 400 px exhibit better accuracy. Figure 4c shows the MAPEs of C11 solved by all seven models when specified percentage (from 0 to 9.6%) of modes were randomly removed. As expected, increasing in mode removal results in increasing of MAPE for all models. The 64 and 144 px dataset-based models exhibit relatively high error than other models (15.0 to 18.2% for 64 px, and 12.0 to 16.4% for 144 px). The models trained by 256 and 400 px dataset exhibit the lowest MAPEs (9.2 to 13.4% for 256 px, and 9.7 to 13.9% for 400 px). Further increasing the resolution of the image-like data does not result in a lower MAPE. Models trained by 576, 784 and 1024 px dataset all show higher MAPE than those by 256 and 400 px, which are about 10.0% when dropping 0% modes, and about 15.0% when dropping 9.6% modes. The C12 solved by the seven models exhibit much better accuracies with lower MAPEs than those of C11, with the trend of MAPEs generally the same (Fig. 4d). The 400 px based model preforms slightly better than the 256 px: MAPEs of 2.1% versus 2.7% at 0% mode dropping, 2.6% versus 2.7% at 2.4% mode dropping, and 3.4% versus 3.7% at 4.8% mode dropping of the test dataset.

We have the following rationale for the impact of the resolution of the image-like data (or modulated fingerprint) on the model performance. Noted that the image-like data obtained from modulation is a reduced form of the raw RUS data, its resolution controls the level of the resulted reduction. Lower resolution gives higher reduction level. Theoretically, the image-like data with infinite resolution contains all information from the raw data, both necessary and unnecessary. Low resolutions, such as 64 and 144 px, yield over-reduction. Some information useful for the NN learning may lose during modulation. To the contrary, high-resolution data (e.g., 576, 784, and 1024 px) carries excessive information that may be unnecessary that can confuse neural network. Data with 256 and 400 px reached a balance between reducing the complexity of the raw data while keeping useful information, making patterns easier to be recognized by NN during model training. Considering that the model trained by 256 px dataset exhibits the lowest validation MSE (see Fig. 4b), we choose this resolution to train all subsequent models.

### Application of modulated fingerprint based NN model for RUS of steel and ceramics

**Figure 5:**
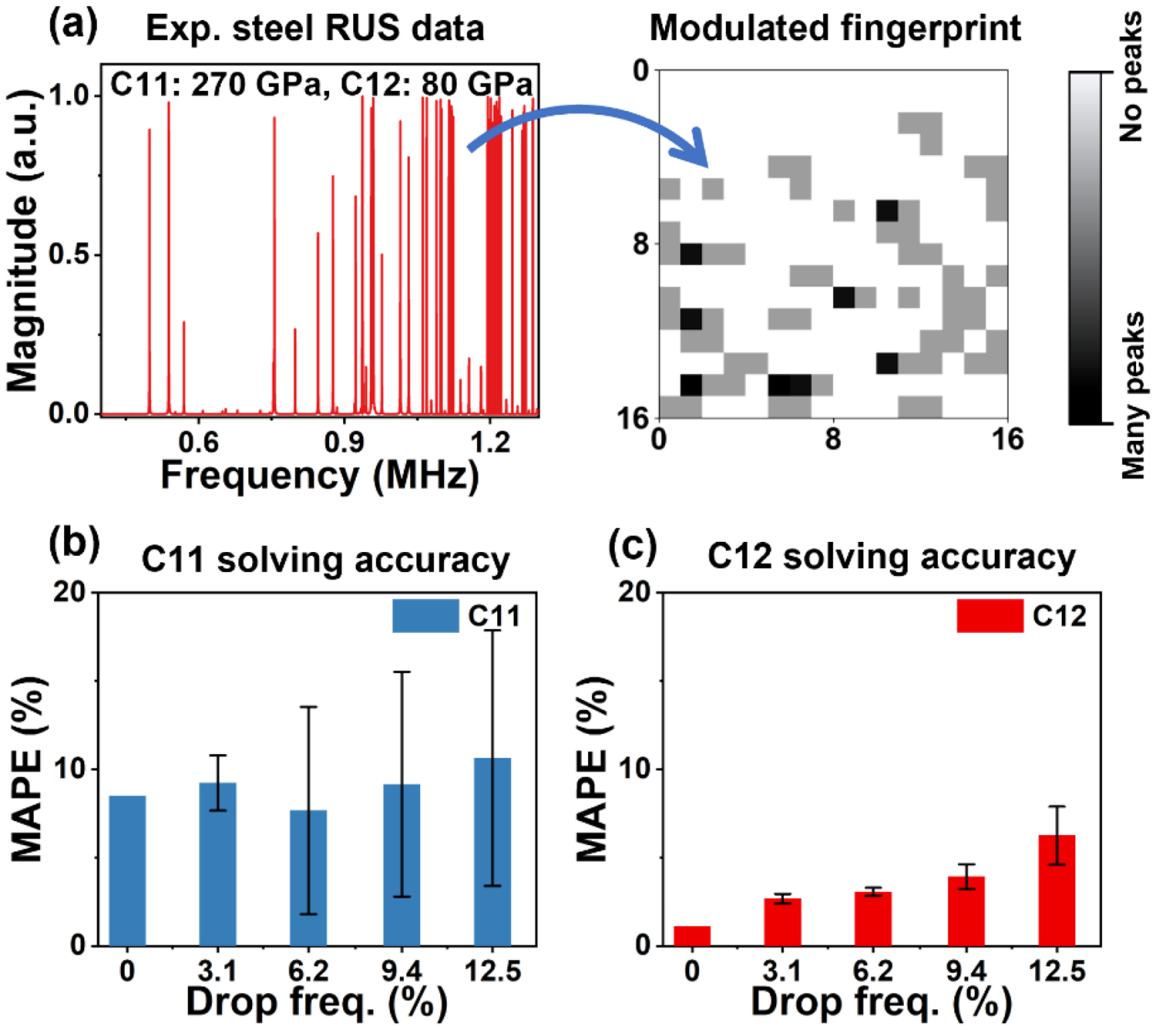
Using neural network model to obtain C11 and C12 from an experimental spectrum of a steel cylinder. (**a**) Experimental steel cylinder RUS spectrum and its corresponding fingerprint. (**b**) and (**c**) Using neural network model to obtain C11(**b**) and C12 (**c**) from experimental data. We randomly dropped specified number of peaks to observe model stability.

Steel has been extensively studied by RUS^61–66^, which provides a good benchmark for testing our fingerprint-based model. We examined the model using a measured spectrum collected from a steel cylinder (see Table S2 for the sample dimension and weight). Before feeding the experimental spectrum into NN model, we manually resolved it and accurately obtained both C11 and C12, as 270.0 GPa and 80.0 GPa, respectively. Missing modes were not observed while resolving this spectrum. As shown in Fig. 5a, we first converted the raw spectrum into the corresponding fingerprint. We randomly removed some resonant frequencies (up to 12.5%) during the fingerprint conversion to simulate missing frequencies. Then, we fed the model with resulting fingerprints and compared the difference between human resolved and NN resolved elastic moduli (C11 and C12, see Table 1) by using MAPE. As shown in Fig. 5b, the absolute error of C11 did not significant increase with the increasing of missing modes, varying between 7.7% and 10.6%. However, the standard deviation surged from 1.5% (missing 3.1% frequencies) to 5.9% (missing 6.2% of frequencies), then gradually increased to 7.2% (missing 12.5% frequencies). The model exhibited higher accuracy and precision while resolving C12 (Fig. 5c). The error was only 3.9% when 9.4% of modes were removed. Only when 12.5% frequencies were removed, the absolute error was significantly increased to 6.2%. Overall, the 256 px fingerprint-based model demonstrated relatively good robustness against missing modes. Moreover, standard deviations for the retrieved C12 (0.3–1.6%) were smaller than those of C11. Although the resolving of C11 may lead to a larger error (about 8.6% or 23.3 GPa compared to manual derivation), it is still in good agreement with the true C11 value. Even values of C11 within the error range provide a good starting point for performing iterations of manual fitting, which greatly accelerates the RUS analysis process by narrowing down the initialization of C11 and C12 from tens of thousands of potential combinations.

**Table 1:** Comparison of resolving elastic moduli from a steel experimental spectrum between human mapping and NN resolving.

Elastic moduli	Human mapping	NN resolved w/ missing peaks				
		0	3.1%	6.2%	9.4%	12.5%
C11 (GPa)	270.0	292.9	294.9 ± 4.2	290.7 ± 15.8	294.7 ± 17.2	298.7 ± 19.5
C12 (GPa)	80.0	80.9	82.1 ± 0.2	82.4 ± 0.2	83.1 ± 0.6	85.0 ± 1.3

**Figure 6:**
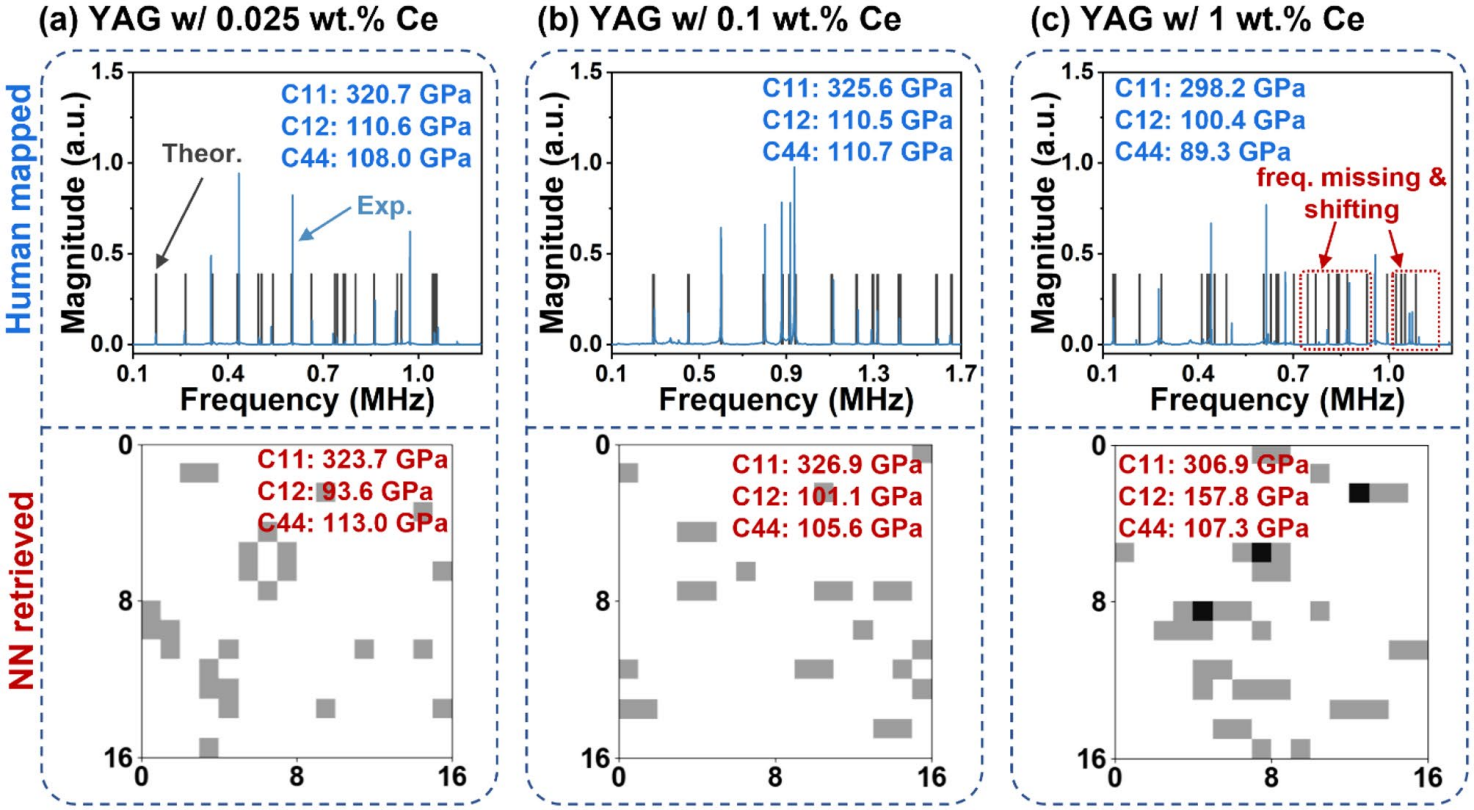
Comparison between human and neural network (NN) model resolved C11, C12 and C44 of YAG ceramic w/ 0.025 wt.% Ce (**a**), 0.1 wt.% Ce (**b**), and 1 wt.% Ce (**c**). The experimental (blue) and theoretical (gray) spectra were overlapped in human resolved figures (upper) to emphasize their difference.

We further applied the modulated fingerprint method to train NN models and resolved three elastic moduli (C11, C12, and C44) from Ce:YAG samples with different amount of Ce (0.025, 0.1 and 1 wt.%). Similar to the steel cylinder, we determined the C11, C12, and C44 range based on previous studies^7,67,68^. In our previous study, we manually resolved the elastic moduli of these Ce:YAG samples and discovered that two samples (0.025 and 0.1 wt.% of Ce) were isotropic^7^, where C44 was the linear combination from C11 and C12 (*C44* = *0.5* × *(C11–C12)*). Ce:YAG with 1 wt.% Ce, being anisotropic, contains three independent elastic moduli. In 0.025 wt.% Ce sample (Fig. 6a), the C11, C12, and C44 previously obtained were 320.7 GPa, 110.6 GPa, and 108.0 GPa, respectively. The NN model retrieved similar values of C11 (323.7 GPa) and C44 (113.0 GPa). The retrieved C12 (93.6 GPa) is slightly different. During the manual mapping, we noticed 26% of modes (10 of 38 modes) were missing by comparing experimental (blue) and theoretical (gray) spectra. Despite such differences, the elastic moduli from NN-based model were closed to those fitted by human. For the 0.1 wt.% Ce sample (Fig. 6b), the NN-based model yielded 326.9 GPa for C11, 101.1 GPa for C12, 105.6 GPa for C44, all in excellent agreement with the human resolved results, 325.6 GPa for C11, 110.5 GPa for C12, and 110.7 GPa for C44. The experimental spectrum from 0.1 wt.% Ce sample is the least distorted among three without any missing modes. Therefore, the NN model solved the elastic moduli with good accuracy.

The human mapped and NN resolved results were more different while analyzing the 1 wt.% Ce sample (Fig. 6c). The only similar elastic modulus was C11 (298.1 GPa vs. 306.1 GPa). C12 and C44 from NN and human were far from each other (100.4 GPa vs. 157.8 GPa for C12, and 89.3 GPa vs. 107.3 GPa for C44). In this sample, the frequency differences between experimental and theoretical spectra were more severe than those in the previous samples. Nearly one third of modes were missing (13 of 42 modes). Some of the remaining experimental resonant frequencies (highlighted by the red boxes) shifted about 3% from their theoretical representation, significantly higher than the distortion we introduced into the training dataset (± 1.5%). The model as expected produced elastic moduli different from those in human determined. Although, the NN-produced moduli are within a “good-guess” range, if one does not know the true values and used them as initialized parameters for performing more rigorous conventional RUS fitting.

The above result showed that the modulated fingerprint-based models (1) tolerated some degree of resonant frequency differences between computed training and experimental data, and (2) successfully retrieved the elastic moduli for 0.025% and 0.1% Ce samples as their final values; and also provide reasonable moduli values for the 1 wt.% Ce sample for further human intervention, significantly lower time and effort to search good initialized moduli sets. Future improvement can be made by using datasets with more variety of theoretical spectra (e.g., higher mode shifting range). Overall, the fingerprint-based models handled both the artificial missing peak (random peak dropping in steel cylinder RUS spectrum), and intrinsic peak differences associated with sample preparation and homogeneity (Ce:YAG RUS spectra). We believe that this modulated fingerprint method is useful to produce high quality NN-based models to retrieve elastic moduli from RUS spectra of various materials.

## Conclusion

In this study, we demonstrated the modulated fingerprint based NN method to be able to (1) preprocess and optimize RUS spectra, (2) tolerate missing or shifting modes, and (3) successfully retrieve elastic moduli either as final values or good initialized ones. We investigated the influence of the fingerprint resolution on the accuracy of the models and found out that 256 px fingerprint-based model exhibited the highest accuracy and robustness in this study. The C11 and C12 MAPEs of 256 px fingerprint-based models were only 10% and 4% when using test spectra with up to 7.2% missing resonant frequencies. Using the optimized model to retrieve elastic moduli from an experimental steel RUS spectrum showed that the model maintained its accuracy. We further examined the modulated fingerprint-based NN method by retrieving elastic moduli from three Ce:YAG samples. The models tolerated up to 26% of missing modes and retrieved both C11 and C12 from two isotropic YAG containing 0.025 and 0.1 wt.% Ce. Applying the NN model on the anisotropic 1% wt. Ce containing YAG sample retrieved C11 accurately, while only providing good guessed values for C12 and C44, due to the severely distorted spectrum containing 30% of missing modes and over 3% of shifting mode frequency. Overall, this study demonstrated that proper data preprocessing and neural network models have the potential of realizing automatic analysis of RUS spectra, which can provide direct solutions or significantly lower time and effort to obtain elastic moduli. Lastly, converting one-dimensional RUS spectra to an image-like modulated fingerprint makes the use of advanced networks, *e.g.*, convolutional neural network, possible in the future studies and transformative in developing deep learning process of other characterization techniques (e.g., XRD, XAFS, mass spectrometry, etc.).

## Data availability

The programs and scripts for generating training dataset, training and evaluating NN models, and analyzing experimental RUS spectra are available at https://github.com/juejing-liu/RUS_TF_GuoGroup under MIT license. Experimental RUS data and models used in this study will be available on request to J.L and X.G.

## Supplementary Information

Supplementary Information.
