# Supplementary material for: A modulated fingerprint assisted machine learning method for retrieving elastic moduli from resonant ultrasound spectroscopy
Source: Sci Rep. 2023 Apr 11;13:5919. doi: 10.1038/s41598-023-33046-w (PMC10090122; doi:10.1038/s41598-023-33046-w)
Supplement: Supplementary file 1 — Supplementary Information. [file 41598_2023_33046_MOESM1_ESM.docx]

**Supporting Information of:**

**A Modulated Fingerprint Assisted Machine Learning Method for Retrieving Elastic Moduli from Resonant Ultrasound Spectroscopy**

Juejing Liu^1,2,3^, Xiaodong Zhao^1,2^, Ke Zhao^2^, Vitaliy G. Goncharov^1,2,3^, Jerome Delhommelle^4^, Jian Lin^5^, Xiaofeng Guo^1,2,3,*^

^1^ *Department of Chemistry, Washington State University, Pullman, WA 99164, United States*

^2^ *Alexandra Navrotsky Institute for Experimental Thermodynamics, Washington State University, Pullman, WA 99164, United States*

^3^ *School of Mechanical and Materials Engineering, Washington State University, Pullman, WA 99164, United States*

*^4^ Department of Chemistry, University of Massachusetts, Lowell, MA 01854, United States*

*^5^ School of Nuclear Science and Technology, Xi’an Jiaotong University, Xi’an, Shaanxi 710049, China*

* e-mail: [x.guo@wsu.edu](mailto:x.guo@wsu.edu)

**Table S1.** Parameters for creating steel and three CeYAG ceramic (0.025%, 0.1%, and 1% Ce) theoretical RUS datasets. All elastic moduli are distributed evenly in the datasets.

| Samples | Parameters | Values |
| --- | --- | --- |
| Steel | Diameter (mm) | 4.76 |
|  | Height (mm) | 3.45 |
|  | Mass (g) | 0.48 |
|  | C11 (GPa) | 170 to 370 w/ 1 increment |
|  | C12 (GPa) | 30 to 130 w/ 1 increment |
| CeYAG w/ 0.025% Ce | Diameter (mm) | 8.62 |
|  | Height (mm) | 1.81 |
|  | Mass (g) | 0.46 |
|  | C11 (GPa) | 270 to 370 w/ 5 increment |
|  | C12 (GPa) | 60 to 160 w/ 5 increment |
|  | C44 (GPa) | 60 to 160 w/ 5 increment |
| CeYAG w/ 0.1% Ce | Diameter (mm) | 4.66 |
|  | Height (mm) | 0.87 |
|  | Mass (g) | 0.07 |
|  | C11 (GPa) | 270 to 370 w/ 5 increment |
|  | C12 (GPa) | 60 to 160 w/ 5 increment |
|  | C44 (GPa) | 60 to 160 w/ 5 increment |
| CeYAG w/ 1% Ce | Diameter (mm) | 8.53 |
|  | Height (mm) | 1.34 |
|  | Mass (g) | 0.31 |
|  | C11 (GPa) | 270 to 370 w/ 5 increment |
|  | C12 (GPa) | 60 to 160 w/ 5 increment |
|  | C44 (GPa) | 60 to 160 w/ 5 increment |

| Sample | Parameter | |
| --- | --- | --- |
| Steel cylinder | Diameter (mm) | 4.76 |
|  | Height (mm) | 3.45 |
|  | Mass (g) | 0.48 |
|  | Frequency range for collecting data (MHz) | 0.2 to 1.3 |
| Ce:YAG w/ 0.025% Ce | Diameter (mm) | 8.62 |
|  | Height (mm) | 1.81 |
|  | Mass (g) | 0.46 |
|  | Frequency range for collecting data (MHz) | 0.1 to 1.2 |
| Ce:YAG w/ 0.1% Ce | Diameter (mm) | 4.66 |
|  | Height (mm) | 0.87 |
|  | Mass (g) | 0.07 |
|  | Frequency range for collecting data (MHz) | 0.1 to 1.7* |
| Ce:YAG w/ 1% Ce | Diameter (mm) | 8.53 |
|  | Height (mm) | 1.34 |
|  | Mass (g) | 0.31 |
|  | Frequency range for collecting data (MHz) | 0.1 to 1.2 |

**Table S2.** Experiential parameters for collecting RUS spectra from steel and Ce doped YAG samples.

* In 0.1% Ce doped YAG sample, we are not able to collect enough resonant peaks between 0.1 to 1.2 MHz


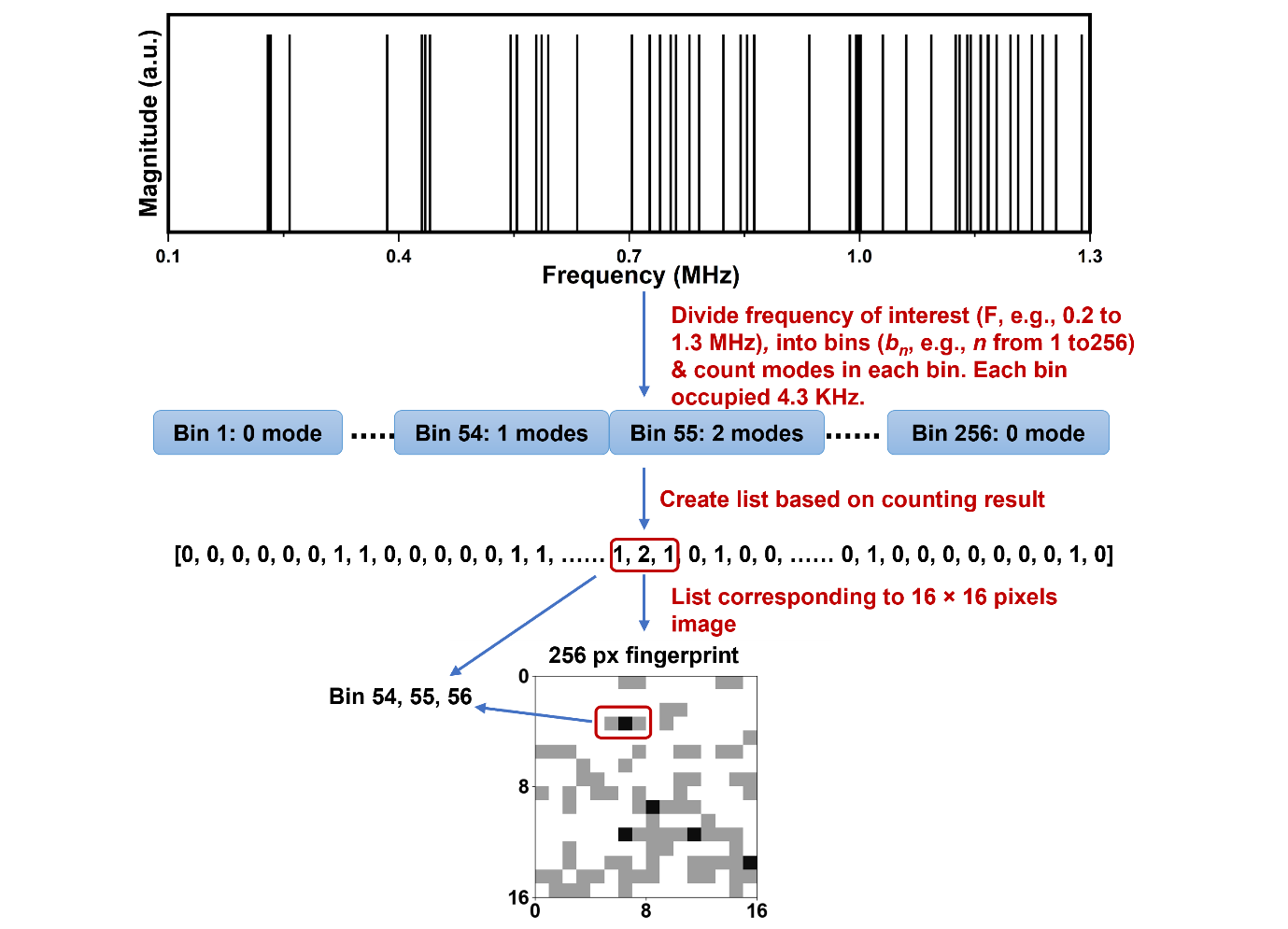


**Figure S1.** Algorithm for converting raw RUS spectrum into image-like modulated fingerprint.

**
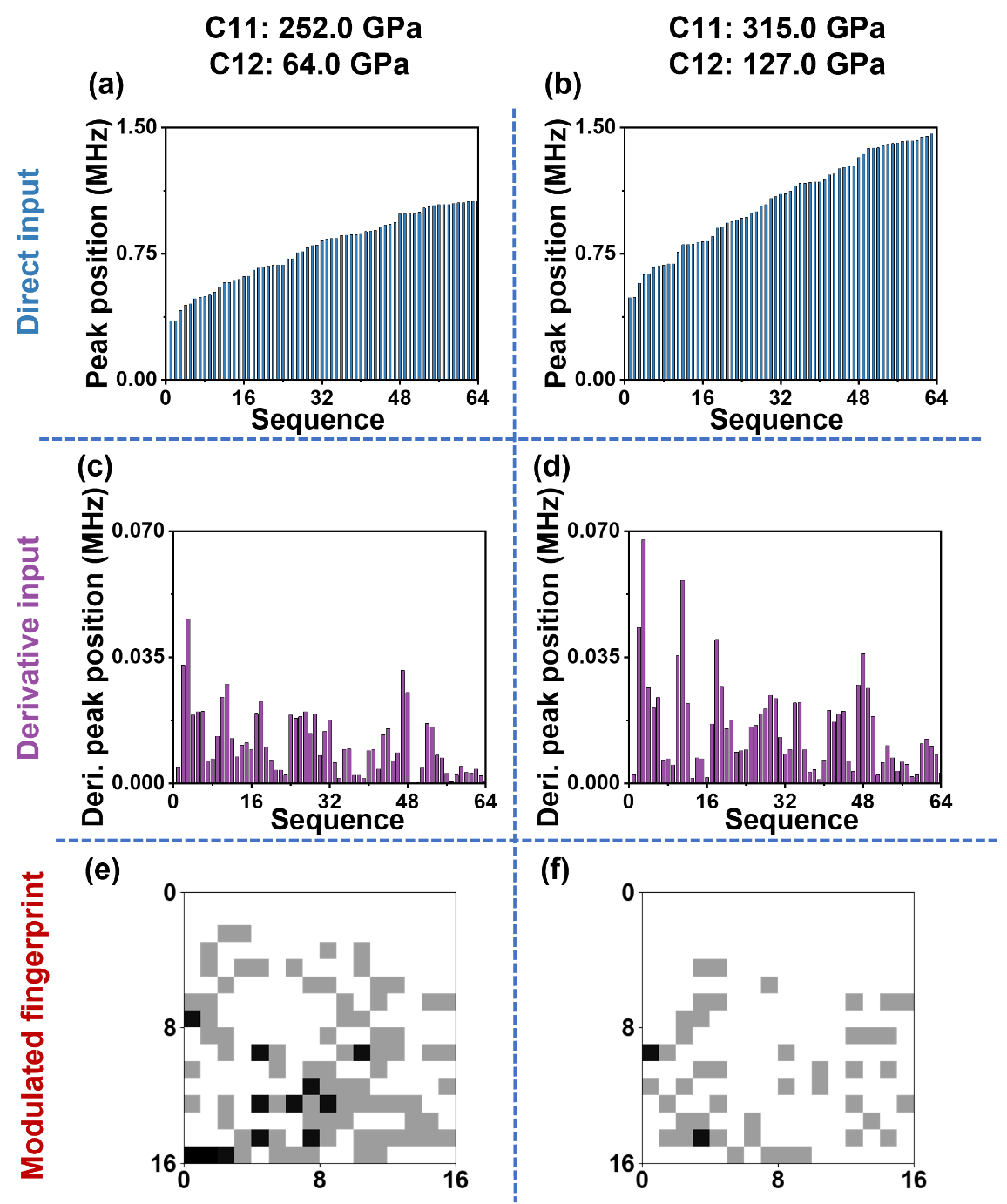
**

**Figure S2.** Two steel theoretical spectra (C11: 252.0 GPa, C12:64.0 GPa and C11: 315.0 GPa, C12: 127.0 GPa) preprocessed by direct input (a and b), derivate input (c and d), and modulated fingerprint (e and f).

**
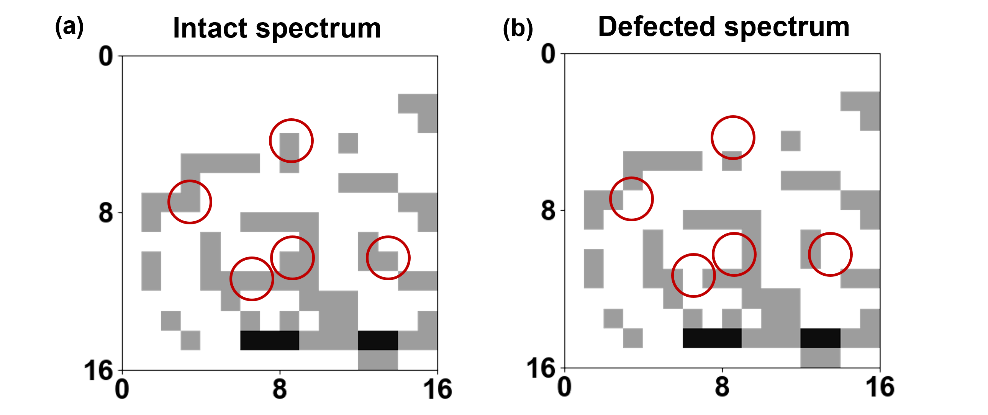
**

**Figure S3. Impact of missing modes on the image-like modulated fingerprint.** A computed steel spectrum with C11 as 268.0 GPa and C12 as 85.0 GPa was used to generate the two fingerprint images. (a) Fingerprint from the intact spectrum. (b) Fingerprint from defected spectrum with 5 missing modes. Differences of two images were highlighted by red circles.

**
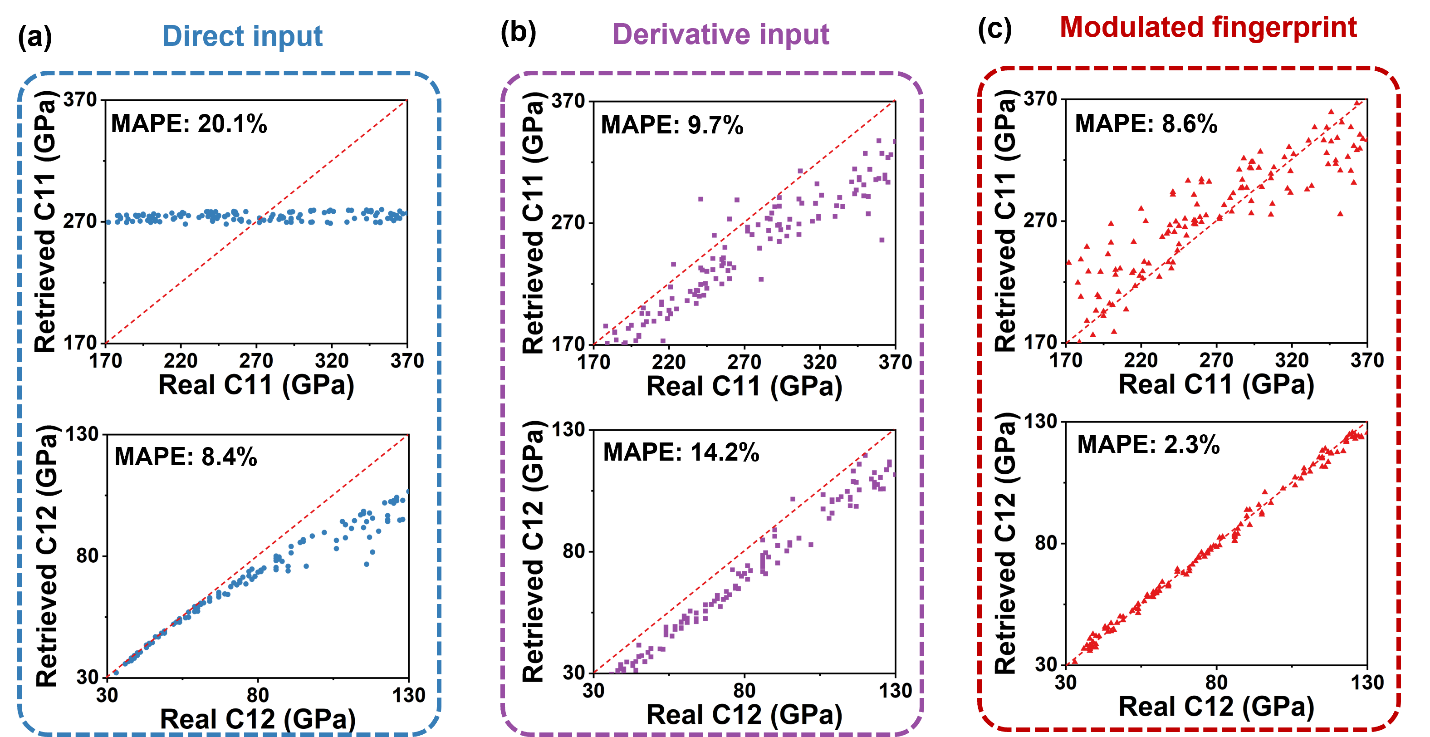
**

**Figure S4. NN resolved elastic moduli from theoretical spectra.** (a) Direct input-based model resolved 128 intact spectra and retrieved C11 and C12. (b) Derivative input-based model resolved 128 intact spectra and retrieved C11 and C12. (c) Modulated fingerprint-based model resolved 128 intact spectra and retrieved C11 and C12.

**
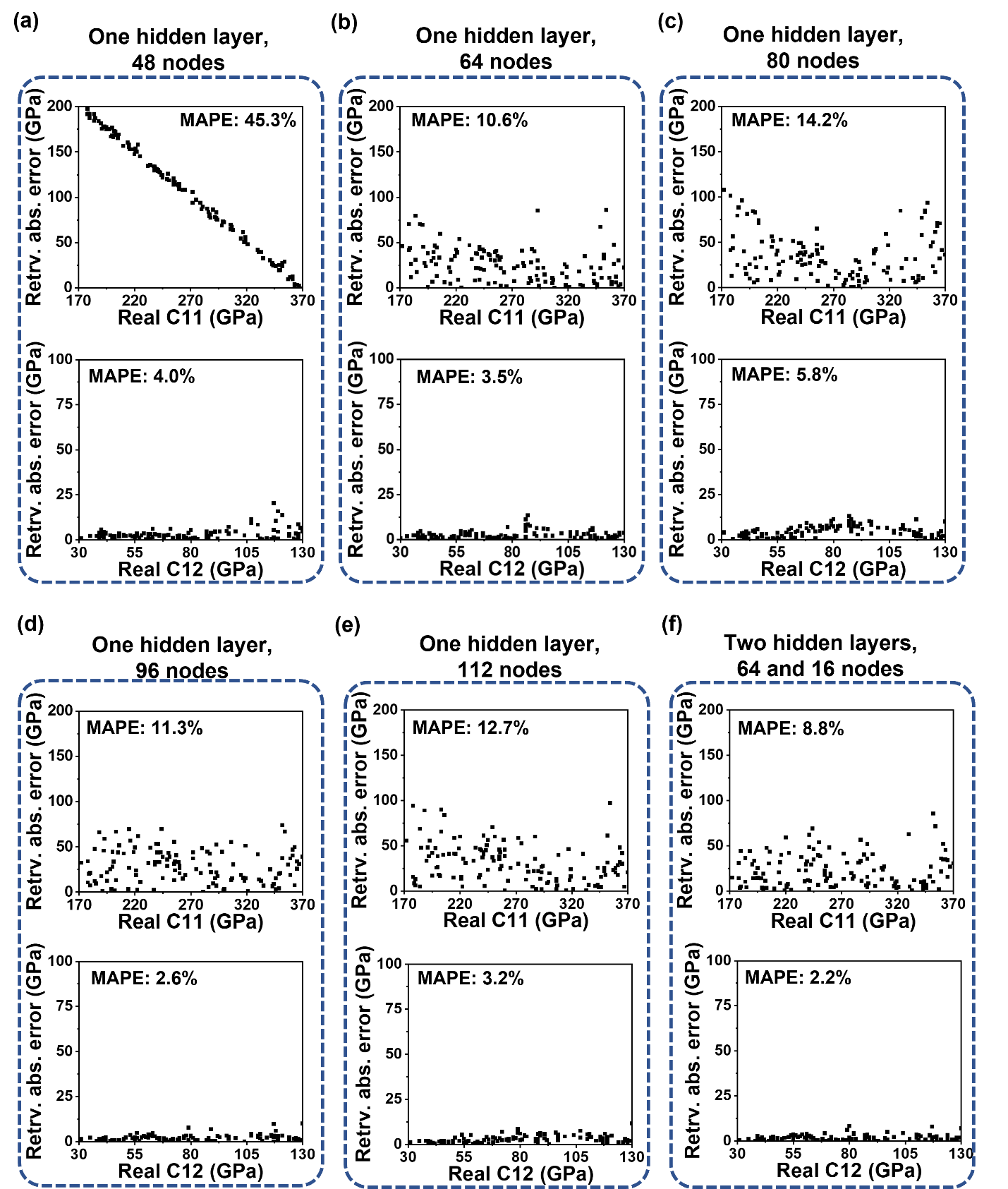
**

**Figure S5. Steel cylinder theoretical spectra C11 and C12 resolving accuracy of modulated fingerprint based NN models with different hidden layers.** 6% of resonant frequencies were removed to simulate missing models. The models consist of one hidden dense layer with 48 nodes (a), 64 nodes (b), 80 nodes (c), 96 nodes (d), 112 nodes (e), and two hidden dense layers with 64 and 16 nodes (f), respectively.

**Table S3. Information density of the image-like datasets with different resolutions.**

| **Resolution (px)** | **Information density (%)** |
| --- | --- |
| 64 | 76.9 |
| 144 | 51.2 |
| 256 | 34.8 |
| 400 | 23.9 |
| 576 | 15.3 |
| 768 | 12.4 |
| 1024 | 9.9 |
